# Interaction and Subcellular Association of PRRT1/SynDIG4 With AMPA Receptors

**DOI:** 10.3389/fnsyn.2021.705664

**Published:** 2021-08-02

**Authors:** Emily Eischen Martin, Erica Wleklinski, Hanh T. M. Hoang, Mohiuddin Ahmad

**Affiliations:** Department of Cell Biology, The University of Oklahoma Health Sciences Center, Oklahoma City, OK, United States

**Keywords:** AMPA receptor, PRRT1, SynDIG4, synapses, phosphorylation, topology

## Abstract

AMPA receptors (AMPAR) are organized into supramolecular complexes in association with other membrane proteins that provide exquisite regulation of their biophysical properties and subcellular trafficking. Proline-rich transmembrane protein 1 (PRRT1), also named as SynDIG4, is a component of native AMPAR complexes in multiple brain regions. Deletion of PRRT1 leads to altered surface levels and phosphorylation status of AMPARs, as well as impaired forms of synaptic plasticity. Here, we have investigated the mechanisms underlying the observed regulation of AMPARs by investigating the interaction properties and subcellular localization of PRRT1. Our results show that PRRT1 can interact physically with all AMPAR subunits GluA1-GluA4. We decipher the membrane topology of PRRT1 to find that contrary to the predicted dual membrane pass, only the second hydrophobic segment spans the membrane completely, and is involved in mediating the interaction with AMPARs. We also report a physical interaction of PRRT1 with phosphatase PP2B that dephosphorylates AMPARs during synaptic plasticity. Our co-localization analysis in primary neuronal cultures identifies that PRRT1 associates with AMPARs extrasynaptically where it localizes to early and recycling endosomes as well as to the plasma membrane. These findings advance the understanding of the mechanisms by which PRRT1 regulates AMPARs under basal conditions and during synaptic plasticity.

## Introduction

AMPA receptors (AMPARs) are ligand-gated ion channels that are activated by neurotransmitter glutamate. They mediate fast excitatory synaptic transmission in the brain and their activity-dependent modulation underlies expression of long-term synaptic plasticity (Malenka and Bear, 2004; Anggono and Huganir, 2012; Huganir and Nicoll, 2013). AMPARs are assembled as homo- or hetero-tetramers of pore-forming GluA1-GluA4 subunits. The subunit composition determines the biophysical properties and trafficking of these receptors (Malinow and Malenka, 2002; Diering and Huganir, 2018). AMPARs are present in supramolecular complexes in which their pore-forming subunits associate with other membrane proteins (Jackson and Nicoll, 2011; Greger et al., 2017; Kamalova and Nakagawa, 2021). The most prominent and well-studied of these transmembrane proteins that act as auxiliary subunits of AMPARs are the transmembrane AMPAR regulatory proteins (TARPs) and cornichon homologs (CNIH-2 and CNIH-3). Extensive work on these proteins has unraveled their profound influence on multiple properties of AMPARs including gating, channel kinetics, deactivation, desensitization and subcellular trafficking (Chen et al., 2000; Tomita et al., 2005a,b; Kato et al., 2010). This work has spurred the development of promising anti-epileptic drugs that target interacting interfaces of AMPARs with specific auxiliary subunits (Maher et al., 2016, 2017; Kato and Witkin, 2018).

Large-scale proteomics investigations on native AMPAR complexes have identified additional associated membrane proteins, which are much less studied than TARPs and cornichon homologs. Proline-rich transmembrane protein 1 (PRRT1) is one such protein that was found to be associated with native AMPARs in multiple studies (Schwenk et al., 2012, 2014; Shanks et al., 2012; Chen et al., 2014). PRRT1 belongs to the SynDIG protein family and alternatively named as SynDIG4 (Kalashnikova et al., 2010; Kirk et al., 2016), as well as to the large Dispanin family which contains other proteins with homologous transmembrane regions (Sallman Almen et al., 2012). We and others have previously investigated the neuronal function of PRRT1 using knockout animals. Deletion of PRRT1 leads to a decrease in surface levels of GluA1 and GluA2 and differentially affects the stability of GluA1 phosphorylated at S845 and S831 sites on the C-terminal tail (Troyano-Rodriguez et al., 2019a). PRRT1 is dispensable for synaptic transmission at mature synapses but is required for single tetanus-induced NMDAR-dependent long-term potentiation (LTP) and NMDAR-dependent long-term depression (LTD) (Matt et al., 2018; Troyano-Rodriguez et al., 2019a). Previous work on PRRT1 expressed in oocytes also identified its role in regulating the biophysical properties of AMPARs (Matt et al., 2018). These studies have elucidated the effect of PRRT1 on AMPARs, however, the mechanism by which PRRT1 regulates these properties is unclear in the absence of information of how PRRT1 interacts with AMPAR subunits. Here we examine the interaction of PRRT1 with AMPARs and identify that it can interact with all AMPAR subunits GluA1-GluA4. We then uncover the membrane topology of PRRT1 and identify the domains that mediate the interaction with AMPARs. Further, we investigate the neuronal subcellular compartments in which PRRT1 might associate with AMPARs and determine the plasma membrane and endosomes as major extrasynaptic sites for their co-localization.

## Experimental Procedures

### Animals

Pregnant Sprague-Dawley rats were obtained from Charles River Laboratories for the preparation of primary neuronal cultures. The animals were provided with water and food *ad libitum*. All procedures on animals were approved by the Institutional Animal Care and Use Committee of the University of Oklahoma Health Sciences Center.

### Plasmids

We amplified cDNA for the generation of various constructs used in this study from mouse brain cDNA. Briefly, mRNA was extracted from pieces of mouse hippocampus and neocortex with RNAqueous-Micro Total RNA Isolation Kit (Thermo Fisher Scientific) according to manufacturer’s instructions. Total mRNA was converted into cDNA with SuperScript VILO cDNA Synthesis Kit (Thermo Fisher Scientific) using random primers. PCR was performed on total cDNA with specific primers (see below) to amplify intended cDNA, which was then cloned into mammalian expression vector L301, in which cDNA is driven by the promoter of human polyubiquitin-C gene (Ahmad et al., 2012). Sequences pertaining to various epitope tags (HA, Flag, and GFP) were added to the cDNA at the indicated positions. Primers used for amplifying cDNA had the following sequence (preceded by appropriate restriction enzyme recognition sites).

PRRT1 forward: ATGTCGTCAGAAAAGTCAGGC
PRRT1 reverse: TTAGGGGTCCCAGTAGTTTTCG
PRRT2 forward: ATGGCAGCCAGCAGCTCTCAG
PRRT2 reverse: TCACTTATACACGCCTAAGTTG
SynDIG1 forward: ATGGATGGCATCATTGAGCAG
SynDIG1 reverse: TCACAGGTGGTTGTTTTTGGAGAGGTA
DSP-B1 forward: ATGGCCAACCCAGCGCAGCCT
DSP-B1 reverse: TTACTTCGGAACTGTGAAATTGA
DSP-C1 forward: ATGGAGAGCCTGAGTGAACTA
DSP-C1 reverse: CTAGCCATGGCCATTCTGGGAC
DSP-C3 forward: ATGGACAACTCCAGCATACAG
DSP-C3 reverse: CTAGGGCGGGTCTCGGGAGGCAAG
PP2B-Aα forward: ATGTCCGAGCCCAAGGCGATTG
PP2B-Aα reverse: TCACTGGATATTGCTGCTATTAC
PP2B-Aβ forward: ATGGCCGCCCCGGAGCCGGCC
PP2B-Aβ reverse: TCACTGGGCACTATGGTTGCC

The following vectors were a kind gift from other investigators: TfR-mCherry (Michael D. Ehlers), PSD95-GFP (Robert C. Malenka), Flag-GluA2 and Flag-GluA3 (Katherine Roche), and Flag-GluA4 (Kari Keinänen).

### Antibodies

The following primary antibodies were used: anti-PRRT1 (ProteinTech, 17261-1-AP, RRID:AB_2878371), anti-PRRT1 (NeuroMab, 75-409, RRID:AB_2531894), anti-GluA1 (Abcam, ab109450, RRID:AB_10860361), anti-GluA1 (NeuroMab, 75-327, RRID:AB_2315840), anti-MAP2 (EnCor, CPCA-MAP2, RRID:AB_2138173), anti-VGLUT1 (Synaptic Systems, 135 303, RRID:AB_887875), anti-EEA1 (Cell Signaling, C45B10, RRID:AB_2096811), anti-Rab7 (Cell Signaling, D95F2, RRID:AB_1904103), anti-Flag (Sigma, F3165, RRID:AB_259529), anti-HA (Thermo Fisher Scientific, 71-5500, RRID:AB_2533988), anti-HA (Biolegend, 16B12, RRID:AB_10064068), anti-GFP (Thermo Fisher Scientific, A-11122, RRID:AB_221569), and anti-β tubulin (Millipore, 05-661, RRID:AB_309885).

The following secondary antibodies were used for immunocytochemistry: Alexa Fluor 488-conjugated, Alexa Fluor 568-conjugated, and Alexa Fluor 647-conjugated goat anti-mouse and goat anti-rabbit immunoglobulins (Thermo Fisher Scientific). The following secondary antibodies were used for immunoblotting: IR800-conjugated goat anti-mouse and goat anti-rabbit (LI-COR); Alexa Fluor 680-conjugated goat anti-mouse and goat anti-rabbit (Jackson Immunoresearch) immunoglobulins.

### Co-immunoprecipitation

HEK293 cells were cultured in DMEM supplemented with 10% fetal bovine serum in a CO_2_-incubator at 37°C. The cells were transfected with calcium-phosphate method 1 day after plating into six-well plates. 24-h later, the cells were collected in lysis buffer (150 mM NaCl, 20 mM Hepes pH 7.4, 0.1 mM EDTA, 2 mM CaCl_2_, 2 mM MgCl_2_, 1% Triton-X100, and protease inhibitor cocktail) and subjected to flip over rotation for 1 h at 4°C. Following centrifugation at 20,000 × *g* for 30 min. at 4°C, the supernatant was collected. The primary antibody was added to the supernatant (at a final concentration of 1–2 μg/ml) and left for flip over rotation overnight at 4°C. Washed and pre-cleared protein A or protein G-agarose beads were added to the supernatant-antibody mix and rotated for 2 h at 4°C. Following centrifugation at 1,000 × *g* for 5 min, beads were washed three times with the lysis buffer (without protease inhibitor). The co-immunoprecipitated proteins were eluted from the beads by adding 2× LDS-containing protein sample buffer (supplemented with 10% β-mercaptoethanol) and heating at 95°C for 5 min. The eluted proteins were then subjected to SDS-PAGE and immunoblotting. Data were collected from three independent experiments.

### Surface Biotinylation

The primary hippocampal cultures or acute hippocampal slices were incubated with 1.5 mg/ml EZ link Sulfo-NHS-SS-Biotin (Thermo Fisher Scientific) on ice for 30 min (cultures) or 45 min (slices) to allow for biotin-labeling of surface proteins. The quenching of unbound biotin was subsequently performed with two washes of 10 min each with 50 mM NH_4_Cl in ACSF (pH 7.4). The cells were homogenized in a lysis buffer containing 150 mM NaCl, 10 mM Tris (pH 7.4), 2 mM EDTA, 1% Triton X-100, 0.5% sodium deoxycholate, 0.1% SDS, protease inhibitor mix (Sigma) and phosphatase inhibitor mix (Roche). The lysate was incubated on ice for 30 min and then centrifuged at 16,000 × *g* for 20 min at 4°C. The supernatant was collected and protein quantification was performed using the BCA assay. To isolate surface proteins, 20 μg of total protein in each sample (for cultures) or indicated amount of protein (for slices) was incubated with 60 μl of NeutrAvidin beads (50% slurry, Thermo Fisher Scientific) for 3 h with flip over rotation at 4°C. The beads were centrifuged at 800 × *g* for 5 min and then washed thrice with lysis buffer that lacked protease and phosphatase inhibitors. The bound proteins on beads were eluted by incubation with 2× sample buffer containing 10% β-mercaptoethanol for 5 min at 95°C. Data were collected from three independent experiments.

### Immunoblotting

The input samples (20 μg protein, corresponding to 2% of total lysate) were mixed with sample buffer containing 10% β-mercaptoethanol and incubated for 3 min at 95°C. The input samples as well as the eluate from beads were loaded on to Bolt Bis-Tris Plus gels (Thermo Fisher Scientific), transferred to nitrocellulose membranes (LI-COR) and immunoblotted with indicated primary antibodies for 2 h at room temperature. After incubation with appropriate secondary antibodies, the membranes were imaged on an Odyssey Imaging System (LI-COR) using the Image Studio acquisition program (LI-COR). The intensity of the bands (sum total of signal in all pixels in the selected area) was quantified using Image Studio Lite (LI-COR).

### Surface Immunostaining

HEK293 cells plated on 12 mm coverslips coated with Poly-L-Lysine were transfected with plasmids using calcium phosphate method. 24 h following transfection, cells were labeled live with primary anti-HA antibody in PBS for 30 min. at 37°C. The excess antibody was washed off with ice-cold PBS and the cells were fixed with 4% paraformaldehyde for 15 min on ice. This was followed by blocking with 3% normal goat serum for 1 h at room temperature and subsequent incubation with secondary antibody in the blocking solution for the same period. In parallel, separate coverslips were used for immunostaining of total HA-tagged proteins by applying primary anti-HA antibody on cells fixed with 4% paraformaldehyde and permeabilized with 0.1% Triton X-100 in PBS for 10 min. The block and secondary antibody application were as described above. The coverslips were mounted on slides in the mounting medium Fluoromount-G (Southern BioTech). The images were acquired with a 63 × 1.4 NA oil-immersion objective mounted on a confocal microscope (Olympus).

### Dissociated Hippocampal Cultures

Dissociated hippocampal cultures were prepared from E18 Sprague-Dawley rats. Hippocampi were dissected out from brains and incubated with a digestion solution containing trypsin for 15 min at 37°C. The tissue was triturated in plating medium containing Neurobasal medium, 2% B27 supplement, 2 mM GlutaMAX and 5% heat-inactivated horse serum (all from Thermo Fisher Scientific). The cells were plated on poly-L-lysine (Sigma) coated glass coverslips placed in 24-well plates at a density of 75,000 cells per cover slip in the plating medium. On the 4th day after plating, the medium was replaced with a maintenance medium containing Neurobasal medium, 2% B27 supplement, 2 mM GlutaMAX and 1% heat-inactivated horse serum. FUDR was added to block glial growth at this time point. Half of the medium in each well was replaced with fresh medium every 3–4 days. Transfection of cultures with TfR-mCherry construct was done at DIV 8–10 using Lipofectamine 2000 (Thermo Fisher Scientific) following the manufacturer’s protocol. The immunocytochemistry experiments were done on cultures at 18–20 DIV.

### Immunocytochemistry on Dissociated Hippocampal Cultures

Eighteen to twenty DIV dissociated hippocampal cultures were fixed with 4% paraformaldehyde (Electron Microscopy Sciences) plus 4% sucrose (Sigma) in phosphate-buffered saline (PBS, Sigma) for 15 min at room temperature, followed by permeabilization with 0.1% Triton X-100 (Sigma) in PBS for 30 min. After blocking with 5% normal goat serum (Jackson ImmunoResearch), the coverslips were incubated with primary and subsequently secondary antibodies at room temperature for 1 h, spaced with multiple PBS washes. The coverslips were mounted on glass slides in Fluoromount-G (Southern Biotech). For co-localization analysis, confocal images were acquired with a 63 × 1.4 NA oil-immersion objective under 2.5× zoom (maintaining Nyquist criterion) with sequential scanning of the same confocal optical section in different channels (Troyano-Rodriguez et al., 2019b). Bleed-through in the optical configuration was checked with control staining in one channel each. 30 μm × 10 μm dendritic segments were isolated from images and thresholded using the Just Another Co-localization Program (JaCoP) in ImageJ. To quantify pixel-to-pixel co-localization in the two channels, Mander’s coefficients M1 and M2 were calculated from images in JaCoP, and multiplied by a factor of 100 to derive the percentage of co-localization. To quantify triple labeling localization (in order to calculate the percentage of co-localized PRRT1 and TfR-mCherry puncta that overlap with GluA1), we constructed an image of overlapping pixels from PRRT1 and TfR-mCherry channels using “AND” operation in Image Calculator in ImageJ. The resultant image was then loaded along with the GluA1 channel image into JaCoP plugin to obtain Mander’s coefficient M1 that represents the fraction of PRRT1/TfR-mCherry co-localized pixels that overlap with GluA1. The brightness and contrast levels of the images were linearly adjusted in ImageJ for presentation in figures. 12–15 dendritic segments were analyzed for each co-localization analysis from three culture preparations.

## Results

### PRRT1 Can Interact With All AMPAR Subunits

**FIGURE 1:**
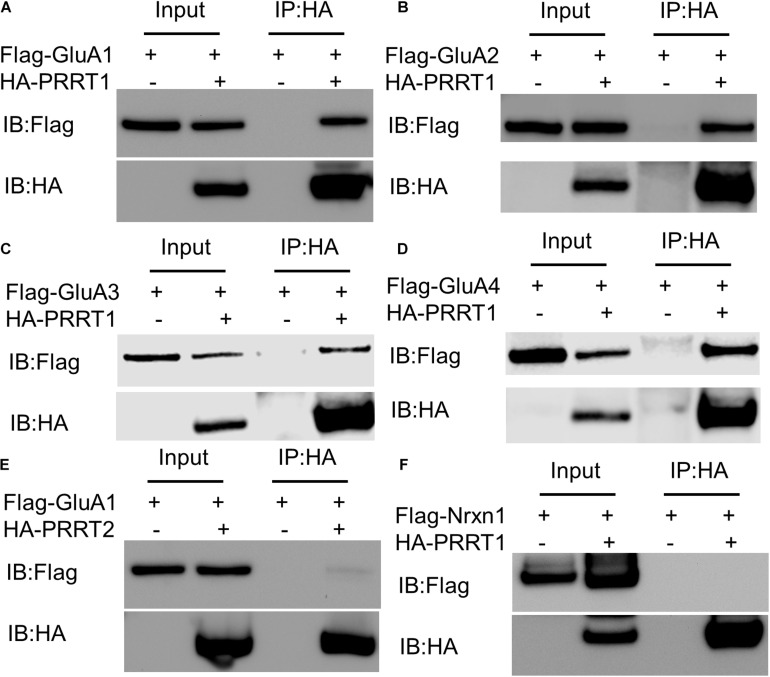
PRRT1 interacts with AMPAR subunits GluA1-GluA4. **(A)** Co-immunoprecipitation (Co-IP) experiments were performed with anti-HA antibody on HEK293 cell lysates expressing Flag-GluA1 and HA-PRRT1. Immunoblotting (IB) of input and immunoprecipitated (IP) samples with anti-Flag (top) and anti-HA (bottom) antibodies shows co-immunoprecipitation of Flag-GluA1 with HA-PRRT1. **(B)** Co-IP of Flag-GluA2 with HA-PRRT1. **(C)** Co-IP of Flag-GluA3 with HA-PRRT1. **(D)** Co-IP of Flag-GluA4 with HA-PRRT1. **(E)** Weak co-IP of Flag-GluA1 with HA-PRRT2. **(F)** No co-IP of unrelated protein Flag-neurexin (Flag-Nrxn1) with HA-PRRT1.

Proline-rich transmembrane protein 1 associates with native AMPAR complexes in various brain regions (Schwenk et al., 2012, 2014; Shanks et al., 2012; Chen et al., 2014). The association of PRRT1 with heteromeric or homomeric AMPAR assemblies could arise due to a direct protein-protein interaction with one or more AMPAR subunits, or indirectly through binding to other components of the complex. In order to understand if there is a physical interaction between PRRT1 and various AMPAR subunits GluA1-GluA4, we performed co-immunoprecipitation experiments from HEK293 lysates expressing PRRT1 and each of GluA1-GluA4 subunits separately. These experiments revealed that PRRT1 co-immunoprecipitates GluA1 (Figure 1A), GluA2 (Figure 1B), GluA3 (Figure 1C), and GluA4 (Figure 1D), indicating that PRRT1 can interact physically with all AMPAR subunits. For comparison, we performed co-immunoprecipitation experiments from HEK293 lysates expressing PRRT2 and GluA1. We observed only weak binding between the two proteins (Figure 1E), which indicates that the association of PRRT2 in AMPAR complexes likely happens through an intermediary protein. No binding of PRRT1 was observed with an unrelated membrane protein Nrxn1 (Figure 1F). The differential binding of PRRT1 and PRRT2 to AMPAR subunits spurred us to expand our analysis to other members of the dispanin family. We amplified the cDNA of the members of dispanin classes B, C and D from mouse brain cDNA (Sallman Almen et al., 2012). We found that the mouse brain expresses DspB1 (Tucs5), DspB3 (PRRT2), DspC1 (SynDIG2), DspC2 (SynDIG1), DspC3 (SynDIG3), and DspD1 (PRRT1). We performed co-immunoprecipitation experiments from HEK293 lysates expressing GFP-tagged dispanin family members and AMPAR subunits. We observed that GluA1-GluA3 pulled down robustly with PRRT1 and DspC1-C3 but not with B-class dispanins PRRT2 and DspB1 (Supplementary Figure 1). These results indicate dispanin class-specific interaction potential of AMPAR subunits.

### Membrane Topology and AMPAR-Interacting Domains of PRRT1

**FIGURE 2:**
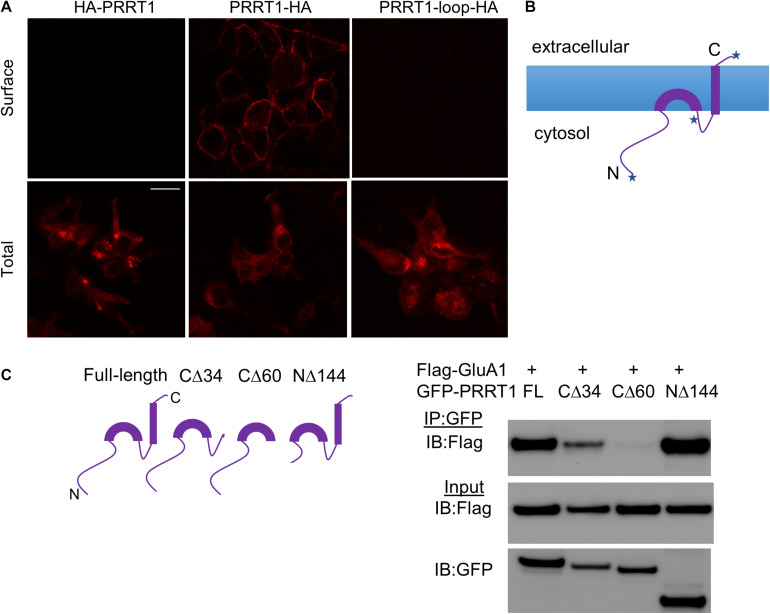
Membrane topology and AMPAR-interacting domains of PRRT1. **(A)** Confocal images of HEK293 cells stained with anti-HA antibody for surface (top panels) or total (bottom panels) PRRT1. PRRT1-HA showed staining on the surface but not HA-PRRT1 or PRRT1-loop-HA. Note that different coverslips were used for surface and total staining. The calibration bar equals 10 μm. **(B)** A cartoon showing the topology of PRRT1 based on the results of surface staining in **(A)**. Each of the three places where HA tag is inserted in PRRT1 is depicted with a star. **(C)** Co-IP experiments were performed with anti-GFP antibody on HEK293 cell lysates expressing Flag-GluA1 and GFP-PRRT1 constructs. Immunoblotting (IB) of immunoprecipitated (IP) samples with anti-Flag antibody (top panel) and of input samples with anti-Flag (middle) and anti-GFP (bottom) antibodies (right). Flag-GluA1 co-immunoprecipitated with GFP-PRRT1 full-length (FL) and NΔ144 but the co-IP with PRRT1-CΔ34 and PRRT1-CΔ60 mutants was weak or absent, respectively. Cartoons of full length and deletion constructs of PRRT1 used in co-IP are shown on the left.

In order to gain further understanding of the interaction of PRRT1 with AMPAR subunits, we first clarified the membrane topology of PRRT1. For this purpose, we made constructs of PRRT1 tagged with hemagglutinin (HA) tag at the N-terminus (HA-PRRT1) and C-terminus (PRRT1-HA). We also placed tandem HA-tags with linkers within the loop (after Q248) between the two predicted hydrophobic, presumably transmembrane segments, of PRRT1 (PRRT1-loop-HA). We then performed surface staining of HA-tag on HEK293 cells expressing these constructs. All three constructs expressed well as evidenced by total HA staining in permeabilized cells (Figure 2A, bottom panels). Robust surface HA staining was observed with PRRT1-HA but not with HA-PRRT1 and PRRT1-loop-HA (Figure 2A, top panels). These results suggest that PRRT1 is oriented in the membrane such that the N-terminus and the loop region of PRRT1 are intracellular while the C-terminus is extracellular (Figure 2B). This topology also suggests that the first hydrophobic segment of PRRT1 does not span the plasma membrane completely while the second hydrophobic segment does (Figure 2B).

In order to map the PRRT1 domains that interact with AMPAR, we made various deletion constructs of PRRT1. In PRRT1-CΔ34 mutant, the second hydrophobic segment predicted in our topology analysis to be the transmembrane domain, was deleted, while in PRRT1-CΔ60 mutant, both the transmembrane domain and the intracellular loop were removed. We also made a PRRT1-NΔ144 construct in which a large part of the intracellular N-terminal domain was deleted. Co-immunoprecipitation experiments in HEK293 lysates revealed that full-length PRRT1 and PRRT1-NΔ144 interacted strongly with GluA1, but weak or no binding was obtained with PRRT1-CΔ34 and PRRT1-CΔ60 mutants, respectively (Figure 2C). These experiments suggest that PRRT1 interacts with GluA1 through its transmembrane region, with contribution from its intracellular loop.

### PRRT1 Co-localizes With AMPARs and Resides at Extrasynaptic Sites on Endosomes

**FIGURE 3:**
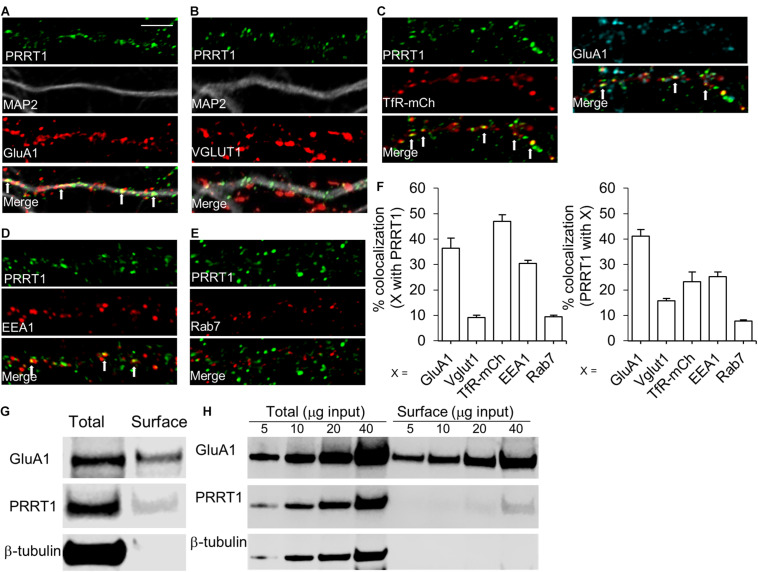
PRRT1 co-localizes with AMPARs and resides at extrasynaptic sites on endosomes. **(A)** Representative confocal images of dissociated hippocampal cultures stained for PRRT1, dendritic marker MAP2 and GluA1. Also shown is an overlay of the three images (merge). PRRT1 (green) co-localizes with GluA1 (red) in the dendrite (gray) at multiple puncta which appear as yellow in the overlay. White arrows in this and subsequent panels point to prominent areas of co-localization. **(B)** PRRT1 (green) does not co-localize well with the excitatory pre-synaptic marker VGLUT1 (red). **(C)** PRRT1 (green) shows robust co-localization with TfR-containing endosomes, labeled with transfected TfR-mCherry (red) as shown in left panels. Co-localization of PRRT1 (green), TfR-mCherry (red) and GluA1 (cyan) is shown in the right panels. **(D)** PRRT1 (green) shows partial co-localization with the early endosome marker EEA1 (red). **(E)** There is minimal co-localization of PRRT1 (green) with the late endosome marker Rab7 (red). **(F)** The left bar graph shows quantification of the percentage co-localization of synaptic and endosomal markers (X) with PRRT1. Right bar graph shows quantification of the percentage co-localization of PRRT1 with synaptic and endosomal markers (X). In all panels, bar graphs represent means ± SEM. The calibration bar equals 5 μm. **(G)** Immunoblots of total or surface fraction following surface biotinylation in hippocampal cultures. GluA1 and PRRT1 are present in surface fraction but not β-tubulin. **(H)** Immunoblots of total or surface fraction following surface biotinylation in acute hippocampal slices. Different amounts of total protein used are indicated. GluA1 and PRRT1 are present in surface fraction but not β-tubulin.

We next investigated the subcellular compartments in which PRRT1 associates with AMPARs. For this purpose, we performed immunocytochemical staining of PRRT1 in cultured hippocampal neurons. Co-immunostaining of cultures with antibodies against PRRT1, AMPAR subunit GluA1 and dendritic marker MAP2 revealed robust co-localization of PRRT1 with GluA1 in the dendrites (Figure 3A). 36.3% ± 4% of GluA1 overlapped with PRRT1, while 41.2% ± 2.6% of PRRT1 co-localized with GluA1 (Figure 3F). There was only a modest co-localization of PRRT1 with the presynaptic marker VGLUT1 as 15.6% ± 0.9% PRRT1 overlapped with VGLUT1 and 9.1% ± 0.9% of VGLUT1 co-localized with PRRT1 (Figures 3B,F). Since these results showed that most of PRRT1 apparently resides outside the synapses but co-localizes well with GluA1, we examined the various extrasynaptic compartments that are involved in AMPAR trafficking. The transferrin receptor (TfR)-containing endosomes provide an important trafficking pathway for AMPARs during constitutive recycling and NMDAR-dependent synaptic plasticity (Ehlers, 2000; Park et al., 2004). To label TfR-containing endosomes, we transfected cultured hippocampal neurons with TfR fused to the fluorophore mCherry (TfR-mCherry) (Wang et al., 2008; Ahmad et al., 2012). Co-immunostaining revealed a robust co-localization of TfR-mCherry and PRRT1, with 46.9% ± 2.6% of TfR-mCherry overlapping with PRRT1, and 23.2% ± 3.8% of PRRT1 overlapping with TfR-mCherry (Figures 3C,F). The latter result is likely an underestimation because analyzed segments unavoidably contained some untransfected neuronal processes. 44.9% ± 8.9% of the co-localized TfR-mCherry and PRRT1 puncta also contained GluA1 (Figure 3C right panels). TfR-containing endosomes can be further categorized into two major sub-populations, namely early endosomes and recycling endosomes (Sonnichsen et al., 2000). We found good co-localization of early endosome marker EEA1 and PRRT1, with 30.4% ± 1.3% of EEA1 overlapping with PRRT1, and 25.3% ± 1.7% of PRRT1 overlapping with EEA1 (Figures 3D,F). In contrast, late endosome marker Rab7 showed little co-localization with PRRT1, with 9.4% ± 0.7% of Rab7 overlapping with PRRT1, and 7.7% ± 0.6% of PRRT1 overlapping with Rab7 (Figures 3E,F). The localization of PRRT1 in TfR-containing endosomes including the early endosomes reveals the key subcellular compartments in which PRRT1 might associate with AMPARs to regulate its trafficking under basal conditions and during synaptic plasticity.

Previous findings from our group and others suggested that PRRT1 might reside on the plasma membrane to stabilize extrasynaptic AMPARs (Matt et al., 2018; Troyano-Rodriguez et al., 2019a). To obtain direct evidence for plasma membrane localization of PRRT1, we performed biotinylation of surface proteins in hippocampal neuronal cultures and acute hippocampal slices. Streptavidin-mediated pulldown of biotin-labeled surface proteins revealed precipitation of PRRT1 (Figures 3G,H), suggesting that a subpopulation of PRRT1 resides at the plasma membrane. As expected, GluA1 was also present in the surface fraction, but there was no signal for β-tubulin, confirming the purity of the surface fraction in our experiments.

### PRRT1 Interacts With Protein Phosphatase PP2B

**FIGURE 4:**
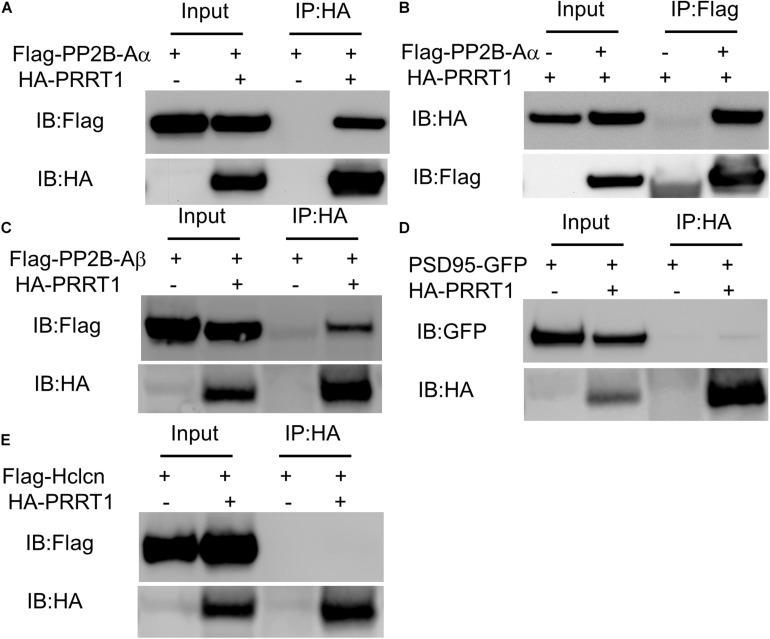
PRRT1 shows interaction with phosphatase PP2B. **(A)** Co-IP experiments were performed with anti-HA antibody on HEK293 cell lysates expressing Flag-PP2B-Aα and HA-PRRT1 constructs. Immunoblotting (IB) of input and immunoprecipitated (IP) samples with anti-Flag (top) and anti-HA (bottom) antibodies shows co-IP of Flag-PP2B-Aα with HA-PRRT1. **(B)** Co-IP of HA-PRRT1 with Flag-PP2B-Aα using anti-Flag antibody for immunoprecipitation. **(C)** Co-IP of Flag-PP2B-Aβ with HA-PRRT1. **(D)** Weak co-IP of PSD-95-GFP with HA-PRRT1. **(E)** No co-IP of Flag-Hippocalcin (Hlcn) with HA-PRRT1.

Previous proteomics studies determined the proteins that associate with PRRT1 in neurons (Chen et al., 2014). In order to elucidate the regulators of PRRT1, we performed experiments to identify proteins that show physical interaction with PRRT1. Previous proteomics work showed an association of PRRT1 with protein phosphatase 2B (PP2B or calcineurin) (Chen et al., 2014), which is a known regulator of GluA1 phosphorylation at serine 845 (S845) (Sanderson et al., 2012; Kim and Ziff, 2014). We reported earlier that hippocampi in PRRT1 knockout mice have altered phosphorylation levels at GluA1 S845 (Troyano-Rodriguez et al., 2019a). To examine if PRRT1 interacts physically with PP2B, we performed co-immunoprecipitation of PP2B catalytic subunit Aα (PP2B-Aα) with PRRT1 expressed in HEK293 cells. Our results revealed a robust pulldown of PP2B-Aα with PRRT1 (Figure 4A). We also performed the experiment in the reverse order, co-immunoprecipitating PRRT1 with PP2B-Aα, and observed good interaction (Figure 4B). Pulldown experiments of PRRT1 with another catalytic subunit PP2B-Aβ also lead to co-immunoprecipitation (Figure 4C). Next, we investigated physical interaction of PRRT1 with PSD-95 and Hippocalcin, two other proteins which were identified as PRRT1-associated proteins in the proteomics study (Chen et al., 2014) and are involved in the expression of NMDAR-dependent LTD (Palmer et al., 2005; Xu et al., 2008). Our co-immunoprecipitation experiments revealed weak or no interaction with PSD-95 and Hippocalcin, respectively (Figures 4D,E), suggesting that these proteins are unlikely to be direct regulators of PRRT1 function or localization.

## Discussion

The results described here decipher the interaction of PRRT1 with AMPAR subunits. Our findings clarify that PRRT1 can interact non-selectively with all AMPAR subunits GluA1-GluA4. Previous work has shown that membrane proteins that associate with AMPARs as auxiliary subunits such as TARPs interact similarly with all AMPAR subunits (Chen et al., 2000; Coleman et al., 2016). In this way, the transmembrane interacting partners of AMPARs may be able to impart broad modulation on all types of AMPARs assembled as homomers or heteromers of different subunits. This contrasts with the selective interaction of some cytosolic proteins with particular AMPAR subunits, imparting subunit specificity to mechanisms of intracellular trafficking of AMPARs (Dong et al., 1997; Srivastava et al., 1998; Malinow and Malenka, 2002). Our co-IP experiments from HEK293 cells which don’t express most neuron-specific proteins also clarify that the association of PRRT1 with GluA1-GluA4 is likely through direct protein-protein interaction (however, a firm conclusion would require determination of protein structures in a complex). Thus, the previously reported effects of PRRT1 on the trafficking and channel properties of AMPARs (Matt et al., 2018; Troyano-Rodriguez et al., 2019a) may involve its direct interaction with the pore-forming subunits. Interestingly, we found that a related homolog PRRT2 interacts weakly or not at all with GluA1-GluA3. This finding is surprising considering that PRRT2 was shown to be part of native AMPAR complexes in the rodent brain (Schwenk et al., 2012, 2014; Shanks et al., 2012). However, the amount of PRRT2 in the native AMPAR complexes is much smaller than PRRT1 (Schwenk et al., 2012, 2014). Also, the principal action of PRRT2 in neurons appears to be the regulation of neurotransmitter release involving its interaction with proteins involved in synaptic vesicle exocytosis including SNAP25, syntaxin 1 and synaptotagmin 1/2 (Lee et al., 2012; Valente et al., 2016; Coleman et al., 2018). These results suggest that PRRT2 works principally at the presynaptic terminal while its minor localization in AMPAR complexes is mediated by an interaction with component/s of the complex other than the pore-forming subunits.

Our surface staining experiments on various tagged constructs deciphered the topology of PRRT1 and revealed that this protein has only one transmembrane domain formed by the second hydrophobic helix. The first hydrophobic helix does not completely span the membrane and either forms a loop on the inner leaflet of the membrane or is peripherally associated with it. Previous attempt to define a complete PRRT1 topology was not successful due to the inability of the loop HA-tagged construct to express (Kirk et al., 2016). We inserted tandem HA tags within the loop between the two hydrophobic segments with appropriate linkers, and found that this construct expresses well in HEK293 cells as evidenced by robust staining in permeabilized cells. Absence of surface staining of this construct thus provided a clear evidence that the loop is intracellular. Interestingly, a similar topology was uncovered recently for PRRT2 (Rossi et al., 2016). However, a different topology has been proposed for SynDIG1 in that the first hydrophobic segment completely spans the membrane while the second one is associated with the extracellular leaflet of the membrane (Kalashnikova et al., 2010). The different topologies of PRRT1 and SynDIG1 suggest that these proteins are unlikely to be close homologs and their regulation of AMPARs may differ mechanistically. Indeed, while the first study on SynDIG1 reported on its effect on regulating AMPAR content at synapses (Kalashnikova et al., 2010), subsequent work has clarified that the principal action of this protein is on the process of synaptogenesis and not on the modulation of AMPARs (Lovero et al., 2013; Chenaux et al., 2016). Interestingly, in the topologies reported previously for Dispanin members PRRT2 and SynDIG1, as well as in the current report on PRRT1, only one of the two hydrophobic segments completely span the membrane. Thus, the name “Dispanin” does not reflect the common membrane topology of the family members and may need to be reconsidered.

Our co-IP experiments with truncated PRRT1 mutants identified that PRRT1 interacts with AMPARs through its transmembrane domain with possible contribution from intracellular loop. These results reinforce the findings that transmembrane interactions constitute a major mechanism by which auxiliary proteins influence AMPARs. It was shown that transmembrane-3 (TM3) and TM4 helices of TARP interact with M1 and M4 of AMPAR subunits (Twomey et al., 2016; Zhao et al., 2016). Similarly, TM1 and TM2 of cornichon-3 interact with GluA subunits (Nakagawa, 2019). TARPs and cornichons modify various properties of AMPARs through these transmembrane interactions including gating and desensitization (Jackson and Nicoll, 2011; Kamalova and Nakagawa, 2021). Thus, the transmembrane interaction of PRRT1 with AMPAR identified here may mediate the reported modulation of AMPAR deactivation and desensitization by PRRT1.

Our immunocytochemical results in primary neuronal cultures show co-localization of PRRT1 with GluA1 in dendrites, with little overlap with excitatory presynaptic marker VGLUT1. These findings are in line with previous evidence of PRRT1 association with AMPARs at extrasynaptic locations (Kirk et al., 2016; Matt et al., 2018; Troyano-Rodriguez et al., 2019a). The strong enrichment of PRRT1 in EEA1 and TfR-containing compartments now identifies major loci at which PRRT1 associates with extrasynaptic AMPARs. The action of PRRT1 at these early and recycling endosomes could help explain the previously published data on the role of this protein in regulating AMPAR trafficking and stabilization. We previously showed that PRRT1 knockout mice have reduced surface expression of GluA1 and GluA2 in the hippocampus (Troyano-Rodriguez et al., 2019a). Since the TfR-containing recycling endosomes act as a source of AMPARs to the plasma membrane (Ehlers, 2000; Park et al., 2004; Wang et al., 2008), PRRT1 at this location may aid in the forward transport. In addition, our surface biotinylation result reported here shows that PRRT1 is also localized to the plasma membrane, where it may serve to stabilize extrasynaptic AMPARs. Thus, in the absence of PRRT1, both forward trafficking of AMPARs from the recycling endosomes to the plasma membrane and the stabilization at the latter may be impaired, leading to the observed impairment of surface GluA1 and GluA2 in the PRRT1 knockout hippocampi. Since the perisynaptic AMPARs are enriched in GluA1 phosphorylated at serine 845 (S845) (He et al., 2009), the lack of stabilization of extrasynaptic AMPARs in the absence of PRRT1 may contribute to the reduced pS845 GluA1 level that was observed in PRRT1 KO.

We also report here a physical interaction between PRRT1 and phosphatase PP2B (calcineurin) catalytic subunits. This finding could be relevant to understanding the mechanism by which PRRT1 regulates AMPAR trafficking under basal conditions and during synaptic plasticity. PP2B is required for NMDAR-dependent LTD and NMDA-induced AMPAR trafficking (Mulkey et al., 1994; Carroll et al., 1999; Beattie et al., 2000), which are impaired in PRRT1 KO mice (Troyano-Rodriguez et al., 2019a). An important substrate for PP2B is GluA1 S845 (Sanderson et al., 2012; Kim and Ziff, 2014), whose basal phosphorylation was reduced in PRRT1 KO animals (Troyano-Rodriguez et al., 2019a). Interaction of PRRT1 with PP2B might stabilize this phosphatase or modulate its activity near AMPARs thus providing a local control of dephosphorylation in the proximity of the receptors. Similar anchoring and regulatory roles have been ascribed to A-kinase Anchoring Protein 150 (AKAP150). Knock-in mice in which wild-type AKAP150 was replaced with a PP2B-interaction deficient mutant showed impaired LTD and elevated basal phosphorylated GluA1 S845 levels, which was explained by the loss of PP2B anchoring close to AMPAR (Sanderson et al., 2012). A decrease in pS845 levels that we observed in PRRT1 KO mice more closely aligns with the possibility that the PRRT1 interaction with PP2B serves to negatively modulate phosphatase activity, similar to the inhibition provided by proteins such as CAIN, RCAN, and FKBP38 (Liu, 2003). Multi-domain proteins like AKAP150 may be more suitable for stabilizing PP2B in AMPAR-containing compartments such as early and recycling endosomes (Purkey et al., 2018). Future experiments will determine the functional significance of PRRT1 interaction with PP2B in regulating AMPAR trafficking.

## Conclusion

Our investigations determine the membrane topology of PRRT1 to be of type II transmembrane protein with a single-pass transmembrane domain formed by the second hydrophobic segment. PRRT1 interacts with all AMPA receptor subunits GluA1-GluA4. The interaction is mediated by the transmembrane domain with contribution from the intracellular loop. We also show that PRRT1 interacts with calcineurin, and resides in the endosomes and plasma membrane at extrasynaptic locations in neurons. While this manuscript was in review, a paper was published on the cryo-EM structure of native hippocampal AMPA receptors in complex with auxiliary subunits (Yu et al., 2021). The structure shows extensive interactions of the transmembrane helix of PRRT1 with an AMPAR subunit, which complements our reported finding here that the transmembrane helix is required for the interaction with AMPARs.

## Data Availability Statement

The raw data supporting the conclusions of this article will be made available by the authors, without undue reservation.

## Ethics Statement

The animal study was reviewed and approved by the Institutional Animal Care and Use Committee of the University of Oklahoma Health Sciences Center.

## Author Contributions

EM, EW, and HH performed the experiments and analyzed the data. MA conceived the study and analyzed the data. All authors contributed to writing of the manuscript.

## Conflict of Interest

The authors declare that the research was conducted in the absence of any commercial or financial relationships that could be construed as a potential conflict of interest.

## Publisher’s Note

All claims expressed in this article are solely those of the authors and do not necessarily represent those of their affiliated organizations, or those of the publisher, the editors and the reviewers. Any product that may be evaluated in this article, or claim that may be made by its manufacturer, is not guaranteed or endorsed by the publisher.
