# Supplementary material for: Interaction and Subcellular Association of PRRT1/SynDIG4 With AMPA Receptors
Source: Front Synaptic Neurosci. 2021 Aug 2;13:705664. doi: 10.3389/fnsyn.2021.705664 (PMC8365426; doi:10.3389/fnsyn.2021.705664)
Supplement: Supplementary file 1 [file Data_Sheet_1.pdf]

Supplementary Material

Interaction and Subcellular Association of PRRT1/SynDIG4 with AMPA receptors

Emily Eischen Martin, Erica Wleklinski, Hanh T.M. Hoang and Mohiuddin Ahmad

Department of Cell Biology, University of Oklahoma Health Sciences Center, Oklahoma City, OK

Running Title: Interactions and Subcellular Localization of PRRT1

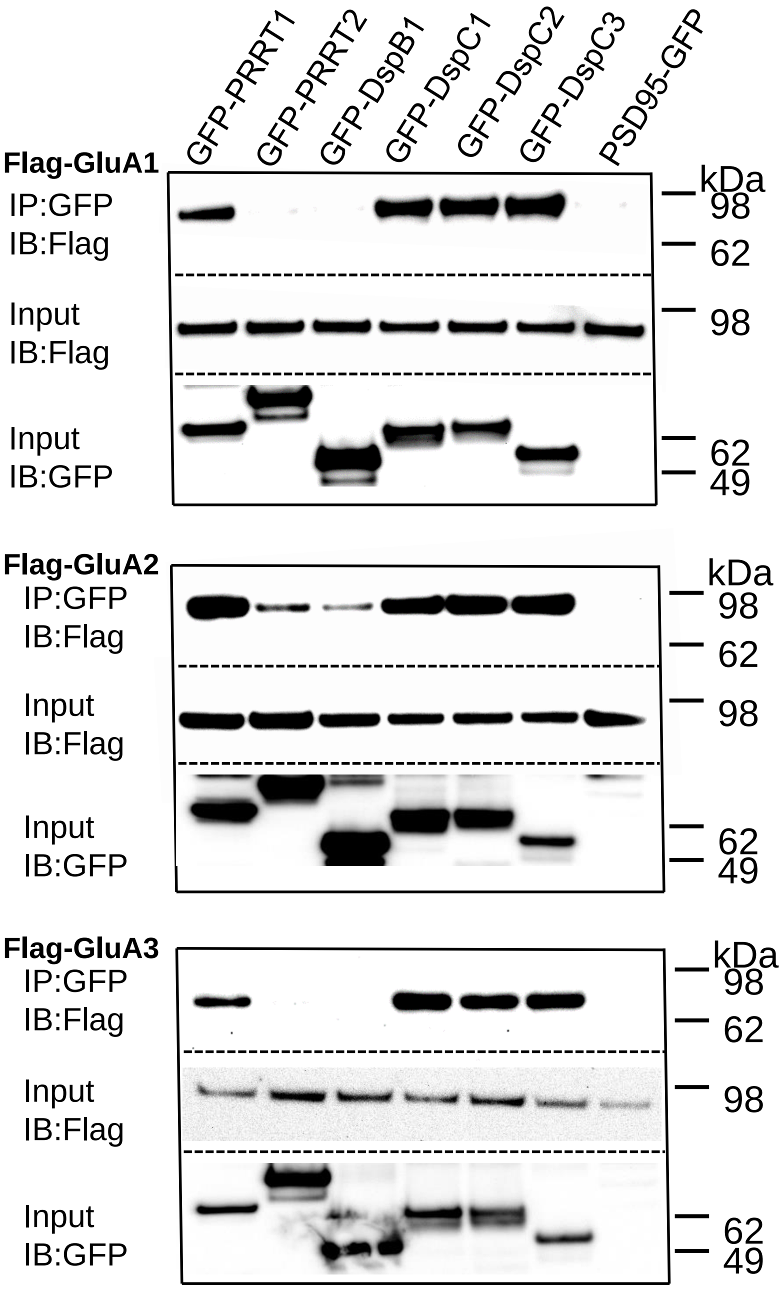

Supplementary Figure 1. Interaction of dispanin family members with AMPAR subunits. Co-immunoprecipitation (Co-IP) experiments were performed with anti-GFP antibody on HEK293 cell lysates expressing Flag-GluA1 (top), Flag-GluA2 (middle) or Flag-GluA3 (bottom) along with the indicated GFP-tagged Dispanin family member. Immunoblotting (IB) of input and immunoprecipitated (IP) samples with anti-Flag and anti-GFP antibodies shows co-immunoprecipitation of Flag-GluA subunits with GFP-tagged Dispanins. PSD95-GFP was used as a negative control (the size of its band in the input was outside the range of the blot shown in the figure).
